# New Insights into Antibacterial Compounds: From Synthesis and Discovery to Molecular Mechanisms of Action

**DOI:** 10.3390/antibiotics9080471

**Published:** 2020-08-01

**Authors:** Jorge H. Leitão

**Affiliations:** IBB—Institute for Bioengineering and Biosciences; Instituto Superior Técnico, Department of Bioengineering, Universidade de Lisboa, Av. Rovisco Pais, 1049-001 Lisbon, Portugal; jorgeleitao@tecnico.ulisboa.pt; Tel.: +351-218417688

**Keywords:** novel antibiotics, antibiotics synthesis, target identification, molecular mechanisms of action, omics approaches in antibiotics research, plant-derived antimicrobials

## Abstract

The worldwide emergence of microbial resistance to available antibiotics presents a global threat to public health and health systems. This special issue aimed to gather papers describing novel antibiotics, originating form chemical synthesis, repurposing of existent drugs, or from natural sources like plant extracts, herbs and spices. A total of 13 papers were published, covering a wide range of topic, including antimicrobial resistance surveillance studies; synthesis of novel molecules with antimicrobial activities; modification or repurposing of already existing molecules, plant-derived active extracts, and molecules; the effects of antimicrobial therapy on microbiota; and the investigation of novel formulations for human and veterinary uses. After decades of antibiotics discovery decline, antibiotics discovery is boosting. Recent developments of post genomics approaches and bioinformatics tools will most certainly turn the tide in the discovery and development of antimicrobials in this exciting field.

The worldwide emergence of microbial resistance to available antibiotics is a threat to public health and health systems at a global scale, leading organizations like the World Health Organization (WHO) to announce a global crisis of antibiotics resistance and to publish a list of priority pathogens in 2017 [1]. Furthermore, old and new pathogens often emerge as global threats, as is the case of the SARS-COV2 coronavirus responsible for the present pandemics. This special issue gathered 13 papers, covering a wide range of topics, including antimicrobial resistance surveillance studies, synthesis of novel molecules with antimicrobial activities, modification or repurposing of already existing molecules, plant-derived active extracts and molecules, the effects of antimicrobial therapy on microbiota, and the investigation of novel formulations for human and veterinary uses.

Surveillance studies carried out to understand how the resistance patterns are evolving in specific groups and regions are critical to understand the trends of antibiotic resistance in both hospital and community-acquired infections and to establish new guidelines for the correct use of existing antibiotics. An example of such studies is illustrated by Folliero et al. in their paper published in this Special Issue, reporting a two-year surveillance study on the antibiotic susceptibility patterns of bacterial pathogens from patients with urinary tract infections attended in a university hospital [2].

The steady increase and spread of resistance to antibiotics among human pathogens has not been accompanied by the introduction in the market of novel antibiotics. The main reasons for the exodus of pharma and biotech companies from antimicrobial research and development rely on the low investment return and demanding regulatory requirements [3]. This lack of investment by pharma and biotech contrasts with the increase of research carried out by both universities and research centers on the search and development of novel antimicrobials. An active area of research encompasses the search for antimicrobial activities of synthetic compounds. Two papers in this special issue present two approaches to optimize the antimicrobial activity of compounds previously described as having antimicrobial activity. The paper by Morsy et al. describes the experimental screening of antimicrobial activities of benzothiazole derivatives against selected bacteria and fungi [4]. The studies were complemented by computer-assisted molecular docking studies, as benzothiazoles were previously demonstrated to inhibit *Escherichia coli* dihydroorotase [4]. In order to gain new insights into the structure/antimicrobial activity relationships, Alves et al. synthesized cyclam derivatives, mono- and trans-disubstituted with benzyl, methylbenzyl, trifluoromethylbenzyl, or trifluoroethylbenzyl moieties appended to the macrocyclic ring nitrogen atoms [5]. The authors conclude that critical parameters for the antibacterial activity of the cyclam derivatives studied include the chemical nature, polarity, and substitution position of the benzyl groups, as well as the number of pendant arms bound to the cyclam backbone [5].

Bacteria remain a useful source of compounds with antimicrobial activity. Terekhov et al. report the use of omics approaches to boost amicoumacin biosynthesis by cultivation under optimized conditions for high-scale production. The compound, active against *Lactobacillales* and *Staphylococcaceae* including methicillin-resistant *Staphylococcus aureus* (MRSA) strains, was previously found by an ultrahigh-throughput screening approach to identify high *Bacillus pumilus* strains producers [6]. Amicoumacin high cytotoxicity and low stability presently hampers its clinical use [6].

The chemical modification of already existing antimicrobials has achieved some success, such as the chemical modifications introduced in the β-lactam ring of penicillin to prevent its hydrolysis by penicillinases. Ying et al. have synthesized aminoglicosides variants containing different alkyl substituents [7]. The authors report that those variants containing C_12_ or C_14_ linear alkyl substituents were serendipitously found to strongly inhibit reverse transcriptase. The authors show evidence that these compounds act by binding nucleic acids with high affinity, forming high-molecular-weight complexes [7]. The compounds were predicted to be useful for nucleic acids localization or in the delivery of nucleic acids to cells or cellular compartments [7].

Repurposing encompasses the finding of novel uses to already approved and available drugs [8]. Various complexes with metal ions like cobalt, platinum, copper, cobalt, zinc, and gold have been described as having anticancer properties [9,10]. One striking example is auranofin, a thiolate-phosphine gold (I) complex initially used to treat rheumatoid arthritis and later found to have antimicrobial activity [11], particularly against *Staphylococcus aureus* [10]. In their article, Alves Ferreira et al. report that one cobalt and two zinc metallic compounds previously found as potential anticancer agents were tested against Gram-positive and Gram-negative bacteria, and they found that one of the compounds was particularly active against *Acinetobacter baumanni* [9], one of the top priority pathogens point out by the WHO [1].

The search for plant extracts and molecules with antimicrobial activities is an intense area of research. Sadgrove et al. describe in their paper the extraction and isolation of seven isoflavone derivatives from *Erythrina lysistemon* bark and the screening of their antimicrobial activity against an extensive panel of bacterial skin pathogens [12]. Results from this study corroborate the traditional use of extracts of *Erythrina* as antimicrobials by demonstrating the antimicrobial activity of isoflavone derivatives as the active principles [12]. In another paper from Sadgrove et al., the antimicrobial and acaricidal activities of terpenes isolated from *Callitris* and *Widdringtonia* (Cupressaceae) were studied [13]. The authors describe, for the first time, new diterpene acids from *Widdringtonia* and compare their chemistry with that of the genus *Callitris* from the Australian clade sister [13]. The paper from Cudalbeanu et al. is the first report of the antifungal, antitumoral and antioxidant properties of the Danube Delta *Nymphaea alba* methanolic extracts [14]. The authors describe the preparation of extracts from leafs and roots of *N. alba* and the evaluation of the above-mentioned properties. Both leaf and root extracts present high fungistatic activity against *C. glabrata* [14]. In addition, the results obtained led the authors to suggest that *N. alba* extracts could be used as an immune booster to prevent infection in immunosuppressed cancer patients [14]. The paper of Phumthum and Sadgrove report the compilation of 125 botanical species used by Karen people from 25 villages in therapies against fever [15]. Various lignans were identified with potential activity as anti-fever agents and as antimicrobials [15].

Antimicrobial peptides (AMP) are promising alternatives for therapeutic interventions to eradicate both human and animal infections. In their paper, Gomes et al. evaluated the antibacterial potential of combinations of 2 AMP, the synthetic pexiganan, and the natural nisin to control the growth of co-cultured diabetic foot clinical isolates of *S. aureus* and *P. aeruginosa* [16]. The authors describe a collagen three-dimensional model of diabetic foot ulcers developed to evaluate both the distribution and antimicrobial efficacy of AMPs [16]. The study evidence the potential of combinations of the 2 AMP to treat difficult-to-eradicate polymicrobial infections associated with diabetic foot ulcers [16]. In another paper from the same research group, Cunha et al. report the study of a Nisin-based gel to control periodontal disease in dogs. This polyphasic study encompassed the evaluation of the effects of dog saliva on nisin antimicrobial activity, the effects of storage conditions on nisin activity, and the cytotoxicity of the nisin gel [17]. The results suggest that the nisin gel is an adequate approach to treat periodontal disease in dogs [17].

The development and validation of antimicrobials also encompass the evaluation of their in vivo efficacy and side effects. In the paper of Kaji et al., the effects of rifaximin on the gut microbiome were investigated [18]. Rifaximin is an antibiotic used to hepatic encephalopathy, a disease comprising a wide range of neurological and psychiatric manifestations [18]. The study was based on 16S rRNA gene sequencing to assess alterations in the microbiota of fecal samples from 30 patients before and after rifaximin treatment [18]. Although the study only gathered a limited number of samples, the results indicate that rifaximin led to minor changes in patients’ intestinal microbiota [18].

After a Golden Age that lasted roughly form the 1930s to the 1950s, antibiotic discovery has declined. The emergence of multi-resistant microbial pathogens, as well as the rise of old and new pathogens like SARS-COV2, poses tremendous challenges to the field of antibiotics discovery and development. The discovery of novel antimicrobials is now empowered with post genomics approaches and bioinformatics tools that will most certainly turn the tide in this exciting field of antimicrobials discovery and development.
